# Carbapenem-resistant bacteria in the environment

**DOI:** 10.2478/aiht-2025-76-3956

**Published:** 2025-06-30

**Authors:** Blanka Dadić, Jasna Hrenović, Tomislav Ivanković

**Affiliations:** University of Zagreb Faculty of Science, Department of Biology, Zagreb, Croatia

**Keywords:** antibiotics, hospital environment, natural environment, One Health approach, resistance, antibiotici, bolnički okoliš, koncept jednog zdravlja, prirodni okoliš, rezistencija

## Abstract

Carbapenem-resistant bacteria (CRB) pose a significant threat to public health due to their resistance to last-resort antibiotics. Even though they are widely studied in clinical settings, much less is known about their presence in environmental compartments with multiple pathways contributing to their dissemination, which raises a growing concern. The aim of this narrative review is to summarise the current knowledge about the occurrence, isolation, and characterisation of CRB in hospital and natural environments and to highlight their clinical relevance and environmental reservoirs. The CRB species pathogenic for humans – *Acinetobacter baumannii*, *Klebsiella pneumoniae,* and *Pseudomonas aeruginosa* – are often identified in hospital and urban sewage, wastewater treatment plants, water bodies, sediments, soil, animals, and plants. Their presence in these environments is largely attributed to anthropogenic factors such as the discharge of untreated or partially treated effluent from wastewater treatment plants. Suitable methods for CRB isolation include selective media, phenotypic assays, and molecular tools for species identification and resistance gene detection. This review also addresses the One Health approach, which stems from the interconnectedness of humans, animals, and environment in the spread of CRB. While the species-level transmission within the One Health framework is well-documented, further research is needed to establish strain-level dissemination patterns. Understanding the mechanisms of CRB persistence and transmission in diverse environments is crucial for developing effective mitigation strategies to curb their spread.

## Abstract (hr)

Bakterije otporne na antibiotike karbapeneme (engl. *carbapenem-resistant bacteria*, krat. CRB) ozbiljna su prijetnja javnom zdravlju zbog svoje otpornosti na antibiotike posljednje linije obrane. Iako ih se opsežno proučavalo u bolničkim okružjima, njihova prisutnost u okolišu postaje sve veći problem, pri čemu višestruki čimbenici doprinose njihovu širenju. Ovaj narativni pregledni rad istražuje prisutnost, izolaciju i karakterizaciju CRB-a u bolničkom okružju i okolišu, ističući njihovu kliničku relevantnost i prirodne rezervoare. Patogene vrste CRB-a, kao što su *Acinetobacter baumannii*, *Klebsiella pneumoniae* i *Pseudomonas aeruginosa*, učestalo se mogu naći u bolničkim i komunalnim otpadnim vodama, postrojenjima za pročišćavanje otpadnih voda, vodnim tijelima, sedimentima, tlu, životinjama i biljkama. Njihova prisutnost u prirodi uglavnom se pripisuje antropogenom utjecaju, kao što je ispuštanje nepročišćenih ili djelomično pročišćenih otpadnih voda. U radu se opisuje i metodologija za izolaciju CRB-a, uključujući upotrebu selektivnih hranjivih podloga, fenotipskih testova i molekularnih alata za identifikaciju vrsta te detekciju gena za rezistenciju. Nadalje, istražen je i koncept jednog zdravlja (One Health), naglašavajući međusobnu povezanost ljudi, životinja i okoliša u širenju bakterija otpornih na antibiotike. Iako je prijenos između različitih bakterijskih vrsta dobro proučen unutar ovoga koncepta, potrebna su daljnja istraživanja kako bi se utvrdili obrasci širenja na razini sojeva. Razumijevanje mehanizama opstanka i prijenosa CRB-a u različitim prirodnim staništima ključno je za razvoj učinkovitih strategija ublažavanja i suzbijanja njihova širenja.

Humankind has already been living in the post-antibiotic era for years (1). The golden era of antibiotics ended in the 1980s, and the emergence and spread of resistance has posed a challenge for many scientists and institutions ever since. In 2017, the World Health Organization (WHO) published for the first time a list of bacterial pathogens that pose risk to human health due to their resistance to several antibiotics (2). This Bacterial Priority Pathogens list has been updated in 2024, following new data obtained from monitoring bacterial infections and deaths (3). The critical group includes carbapenem-resistant *Acinetobacter baumannii* (CRAB) and carbapenem-resistant *Enterobacterales* (CRE). These high-priority bacterial species are resistant to carbapenems, a class of antibiotics in the last line of defence against infections caused by multi-drug resistant bacteria (4) characterised by the beta-lactam ring in their chemical structure. According to the 2023 European Centre for Disease Prevention and Control report, 18 % of European countries reported a ≥50 % carbapenem resistance rate for *Klebsiella pneumoniae* and a 56 % rate for *Acinetobacter* spp. (5). Cases of CRAB are more common in the southern parts of Europe. For example, Croatia has seen a significant increase since 2008, with the resistance rate to imipenem and meropenem reaching 96 % by 2023 (6).

For these reasons, research of carbapenem resistance has intensified over the last decade and has addressed the issue of the linkage between the global spread of CRAB and CRE species in hospitals, healthcare facilities, and the environment. Although numerous studies have confirmed the presence of carbapenem-resistant bacteria (CRB) in the environment, the entry points and pathways of their spread remain unknown. The risk of infection increases with potential transmission between humans, animals, and the environment, but its mechanisms have not been confirmed due to insufficient research data (4). The spread of infectious diseases and antimicrobial-resistant genes between humans, animals, and the environment is investigated through the One Health approach (7). International organisations, such as the World Organisation for Animal Health and the United Nations WHO and Food and Agriculture Organization, have developed a plan to monitor the occurrence of antimicrobial resistance and the use of antibiotics in order to gain insights into the possible connection (8).

This narrative review lists the most frequent CRB strains in the environment, describes the main characteristics of carbapenem resistance mechanisms, and the methods for isolating and characterising CRB species from the environment. Furthermore, it provides a broader view of possible CRB reservoirs, such as hospital environment, hospital and urban sewage, wastewater treatment plants (WWTP), animals, plants, and the natural environment.

## CLINICAL IMPORTANCE OF CARBAPENEM-RESISTANT BACTERIA

The most common CRB infections in humans are caused by *A. baumannii*, *K. pneumoniae*, and *Pseudomonas aeruginosa*. These species belong to the group of so called ESKAPE pathogens (*Enterococcus faecium*, *Staphylococcus aureus*, *K. pneumoniae, A. baumannii, P. aeruginosa*, and *Enterobacter* spp.), which are the leading antibiotic-resistant species worldwide (9).

*A. baumannii* is an opportunistic pathogen that colonises the mucous membranes of the respiratory system and injured skin (10, 11). It can cause pneumonia (both hospital- and community-acquired), bacteraemia, and soft tissue and wound infections (12). It is the most common cause of nosocomial infections and presents a particular risk for patients in intensive care units, especially if treatment requires the use of urinary or intravascular catheters and respirators (13). Hospital spread of infections is facilitated by improper maintenance and hygiene of equipment, as certain strains can survive on various surfaces for long. *A. baumannii* has been reported to survive for 90 days on dry cotton (14). CRAB is the most important species in clinical settings. However, some studies have isolated carbapenem-resistant non-baumannii *Acinetobacter* spp. from clinical samples. For example, Park et al. (15) found that 17 of 160 tested non-baumannii *Acinetobacter* spp. were resistant to imipenem or meropenem.

*K. pneumoniae* is another opportunistic pathogen that colonises the mucous membranes of the respiratory and digestive systems and can cause pneumonia, sepsis, and urinary infections (16, 17). According to the 2022 report by the European Antimicrobial Resistance Surveillance Network, 97.1 % of *K. pneumoniae* isolates detected in the European Union and European Economic Area countries were resistant to carbapenems (18).

*P. aeruginosa* causes chronic pneumonia in immunocompromised individuals suffering from cystic fibrosis, cancer, or ventilator-associated pneumonia (19, 20). Ten strains are considered high-risk clones, as they produce extended-spectrum beta-lactamases and carbapenemases, including the ST235, ST111, ST233, ST244, ST357, ST308, ST175, ST277, ST654, and ST298 (19, 21). Among them, the ST235 stands out, as it produces several different carbapenemases (21).

All three CRB species cause nosocomial infections, primarily in immunocompromised patients, and are therefore a serious problem in hospitals. Patients in intensive care units undergo various invasive procedures or require different devices (such as ventilators or catheters), which put them at high risk of infection, and infections caused by CRB species pose a big treatment challenge because of their resistance to various other classes of antibiotics and, if left untreated, can lead to sepsis, multi-organ failure, and eventually death. In 2017, the US Centers for Disease Control and Prevention (CDC) reported 13,100 cases of infections with CRE and 8,500 with CRAB in and out of hospitals (22). There are several treatment options that use last-resort drugs or newly approved antibiotics in mono- or combined therapy. The treatment of CRE infections often includes a combination of tigecycline with colistin and aminoglycosides, whereas CRAB infection treatment relies on a combination of minocycline and cefoperazone-sulbactam, tigecycline and amikacin, or high doses of ampicillin-sulbactam with polymyxin B (23). In addition, colistin is frequently used as the last-line treatment, but resistance has already been reported in some clinical isolates (24). Among the newly approved antibiotics, there is cefiderocol, which has demonstrated activity against *P. aeruginosa*, *A. baumannii*, OXA-48, and *K. pneumoniae* carbapenemase (KPC) producing CRE (23, 25).

## ISOLATION AND IDENTIFICATION OF CARBAPENEM-RESISTANT BACTERIA FROM THE ENVIRONMENT

### Cultivation methods

**Figure 1:**
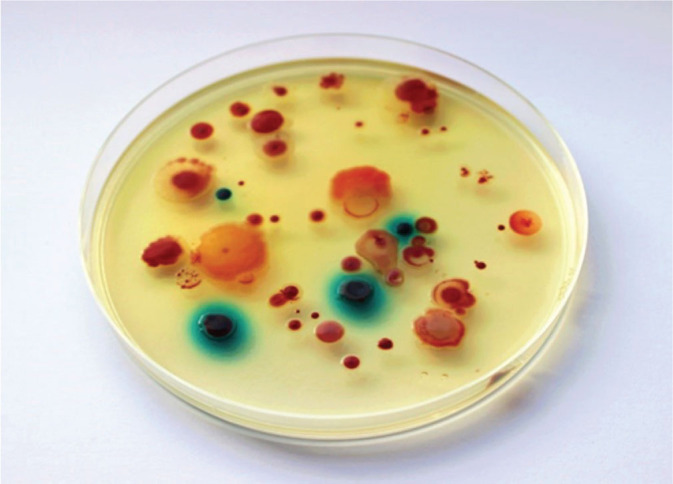
Carbapenem-resistant bacterial species from river sediment cultured on CHROMagar Acinetobacter. Red – *Acinetobacter* spp., *Pseudomonas* sp., and *Stenotrophomonas* sp. (similar to *Acinetobacter* but of different morphology). Blue – *Enterobacterales*

Cultivation of CRB by using nonselective microbiological media is uncommon, because CRB are overgrown by autochthonous bacteria or physiological flora. Nowadays, there are various commercially available selective media used for the detection of CRB from environmental samples such as water, soil, or sediment. CRE are widely cultivated in chromogenic media such as Brilliance CRE (Oxoid, Wesel, Germany), chromID CARBA (bioMérieux, Nürtingen, Germany), Chromatic CRE (Liofilchem, Roseto degli Abruzzi, Italy), chromID OXA-48 (bioMérieux, Nürtingen, Germany), and CHROMAgar mSuperCARBA (CHROMAgar, Paris, France) (26, 27). Chromogenic media contain enzyme substrates linked to a chromogen substance that gives a specific colour reaction to a specific bacterial colony. Brilliance CRE can be used for optimal detection of CRE, while chromID OXA-48 and CHROMAgar mSuperCARBA are suitable for detecting OXA-48-producing strains (27, 28). Selective media have been designed to allow the growth of other CRB such as *Acinetobacter* sp., *Pseudomonas* sp., *Stenotrophomonas* sp. and *Klebsiella* sp. An example of such a medium is CHROMagar Acinetobacter with the addition of CR102 supplement (CHROMagar, Paris, France) (29) (Figure 1).

Extended-spectrum beta-lactamase (ESBL) screening media can also be used, but their major disadvantage is that ESBL-producing bacteria can overgrow small CRB cultures (28). Apart from chromogenic media, many studies use media supplemented with carbapenems to achieve selectivity for CRB growth. One such medium is MacConkey agar, often used because of its selectiveness for gram-negative bacteria. For example, Tacao et al. (30) determined 19 CFU/mL of CRB in river water by comparing the count of resistant bacteria cultivated on MacConkey agar spiked with 8 µg/mL of imipenem with the count of susceptible strains (980 CFU/mL) in the same samples cultivated on MacConkey agar without imipenem. A similar method was used with MacConkey agar supplemented with ertapenem (31).

Besides the appropriate selective medium, culture incubation temperature can play a crucial role in differentiating between CRB with intrinsic and acquired resistance. This can be achieved with a simple protocol in which samples are incubated in parallel at temperatures of 37 °C and 42 °C (32). Incubation at 37 °C allows the growth of bacteria with acquired antibiotic resistance but also the growth of bacteria with intrinsic resistance to carbapenems, such as *Stenotrophomonas* sp. and *Aeromonas* spp. (33, 34). Incubation at 42 °C, however, is a screening threshold at which only bacteria with acquired resistance can grow, whereas the growth of intrinsically resistant bacteria is inhibited. This observation is consistent with the reported counts of CRB from a stream, lake, well, and landfill leachate grown at 37 °C, which were 1.0, 4.6, 1.3, and 44 CFU/mL, respectively (29). CRB did not grow at 42 °C in the first three samples, but their count in leachate was 25 CFU/mL, which was associated with untreated hospital wastewater as a source of clinically relevant species. This protocol is not applicable for long-term monitoring of bacterial survival due to a possible loss of thermotolerance in the presence of antibiotics. Namely, Dekić Rozman et al. (35) reported that after a prolonged incubation in sterile spring water, *A. baumannii* could no longer grow with carbapenems at 42 °C but could at 36 °C. The authors explained this with nutrient starvation in sterile water, which may have altered the structure of the cell membrane to such extent that elevated temperature, which affected outer membrane proteins, increased membrane permeability to carbapenems.

### Identification of carbapenem-resistant bacteria

Once grown on agar plates, pure culture isolates of suspected CRB need to be identified with relative certainty. One method that does that is the matrix-assisted laser desorption ionisation coupled to time-of-flight mass spectrometry (MALDI-TOF MS). The method is quick and simple, and species identification is based on the analysis of the bacterial proteome (36). In addition, based on protein biomarkers, it can differentiate between closely related strains (37). In a study by Hrenović et al. (38) this method confirmed the presence of *K. pneumoniae* in river water, cultured on a selective media and determined with a VITEK^®^ 2 system (bioMérieux, Lyon, France). MALDI-TOF MS also proved reliable in identifying *Aeromonas* spp. (39, 40). Apart from identification, MALDI-TOF MS can detect carbapenemase activity but cannot specify the type (41). For example, it showed complete meropenem degradation by two wastewater isolates of *A. baumannii* over 2.5 h through characteristic peaks at m/z 379, 401, 423, and 445 in the spectrum (33).

The usual molecular method to identify bacterial species is 16S rRNA gene sequencing. For example, Henriques et al. (42) partially sequenced the 16S rRNA region of isolates from untreated drinking water and identified bacterial species from the following genera: *Stenotrophomonas*, *Pseudomonas*, *Janthinobacterium*, *Ralstonia*, *Acidovorax*, *Cupriavidus*, *Caulobacter*, and *Sphingomonas*.

Common molecular methods that can identify genes encoding carbapenemases are polymerase chain reaction (PCR) and quantitative PCR (qPCR). Using the TaqMan multiplex qPCR, Oliveira et al. (43) successfully identified the *bla*_KPC_, *bla*_OXA-48_, and *bla*_VIM_ genes in samples isolated from a WWTP.

Whole genome sequencing (WGS), multilocus sequence typing (MLST), and core genome MLST (cgMLST) provide information about genome size, the number and type of plasmids, genes involved in resistance mechanisms, and the strain sequence type. In one study (44), MLST determined the sequence type 11 (ST11) in *K. pneumoniae* isolated from estuary river water based on the PubMLST scheme. Knowing the strain sequence type helps to follow the spread of clinical isolates into the environment and establish the relationship between environmental and clinically relevant isolates. Kehl et al. (45) used cgMLST to determine the connection between hospital isolates and isolates determined in urban WWTP. All the PubMLST scheme ST147 *K. pneumoniae* strains formed a single epidemiological cluster and the results indicated that clinical isolates spread directly into the environment. WGS can be used for additional confirmation of the species type following 16S rRNA sequencing as well as for the detection of genes responsible for carbapenem resistance.

Metagenomic analysis is a method that enables direct sequencing of genetic material from environmental samples without prior isolation and cultivation of CRB species. Makowska et al. (46) used it to evidence the presence of carbapenem resistance genes (*bla*_NDM_, *bla*_VIM_, *bl*a_GES_, *bla*_OXA-48_) in the final effluent from a WWTP.

### Phenotypic characterisation of carbapenem resistance in environmental isolates

**Figure 2:**
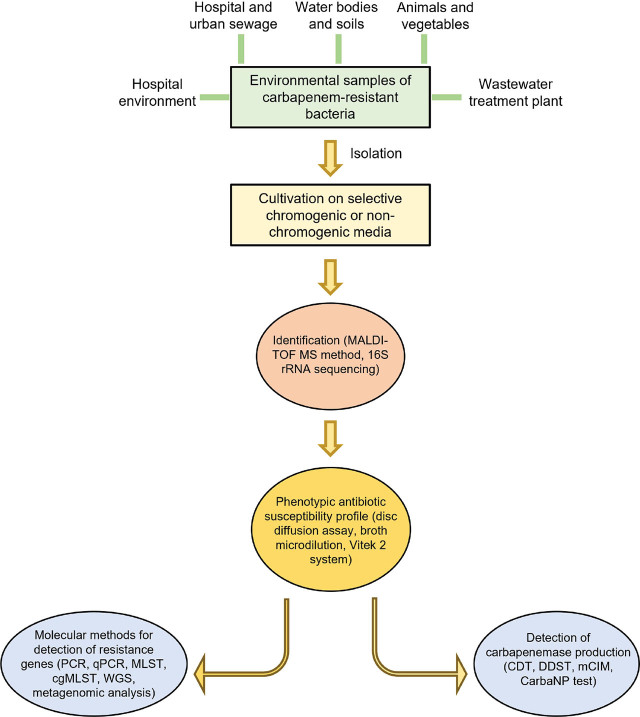
Recommended protocol for the detection and characterisation of CRB from the environment

After cultivation on the medium, the obtained species should be tested for susceptibility to carbapenems and beta-lactam antibiotics using either broth microdilution, disk diffusion, or the bioMérieux VITEK^®^ 2 system (Figure 2) (26). Due to the lack of criteria for environmental isolates, the cut-offs for susceptibility, expressed as minimum inhibitory concentrations (MICs), are taken from the standards used for clinical isolates, such as the European Committee on Antimicrobial Susceptibility Testing (EUCAST), common in the EU, or those issued by the Clinical and Laboratory Standards Institute (CLSI) in the USA (47, 48). Both standards are recommended by the WHO’s global Antimicrobial Resistance Surveillance System (49). EUCAST and CLSI MICs for carbapenems differ slightly, and both standards exclude ertapenem against *Acinetobacter* spp. and *Pseudomonas* spp., as ertapenem is unsuitable for treatment with these two species (Table 1).

**Table 1:** Minimum inhibitory concentrations (MIC) of imipenem, meropenem, and ertapenem according to EUCAST (48) and CLSI (49) criteria for clinically relevant CRE, CRA, and carbapenem-resistant *P. aeruginosa*

	**MIC values (mg/mL)**					
	***Enterobacterales***		***Acinetobacter* spp.**		***Pseudomonas* spp.**	
	**EUCAST**	**CLSI**	**EUCAST**	**CLSI**	**EUCAST**	**CLSI**
Imipenem	S≤2	S≤1	S≤2	S≤2	S≤0.001	S≤2^2^
	R>4	R≥4	R>4	R≥8	R>4	R≥8^2^
Meropenem	S≤2^1^	S≤1	S≤2^1^	S≤2	S≤2^1,2^	S≤2^2^
	R>8^1^	R≥4	R>8^1^	R≥8	R>8^1,2^	R≥8^2^
Ertapenem	S≤0.5	S≤0.5	-	-	-	-
	R>0.5	R≥2	-	-	-	-
Doripenem	S≤1	S≤1	S ≤0.001	S≤2	S≤0.001	S≤2^2^
	R>2	R≥4	R>2	R≥8	R>2	R≥8^2^

1 The MIC values refer to indications other than meningitis;

2 The listed values apply only for *P. aeruginosa*;

R – resistant; S – sensitive

In addition to phenotypic antibiotic susceptibility profiling, it is necessary to determine the underlying mechanism of carbapenem resistance for epidemiological purposes. The most common methods for that are the combined disc test (CDT), double-disc synergy test (DDST), modified carbapenem inactivation method (mCIM), and the Carbapenemase Nordmann-Poirel (CarbaNP) test (26, 50, 51). CDT combines a disc containing the antibiotic and a disc containing the antibiotic and a beta-lactamase inhibitor, while the DDST keeps the inhibitor and antibiotic separate (26). A strain tested with the CDT is considered positive for carbapenemases if the disk containing both the antibiotic and the inhibitor yields an inhibition zone of 5 mm or is 50 % larger in diameter than the disc containing only the antibiotic (52). The DDST is considered positive if the inhibition zone between the disc containing the antibiotic and the disc containing the inhibitor is larger than the inhibition zone on the other side of the discs (53). Inhibitors used in these tests are ethylenediaminetetraacetic acid (EDTA), faropenem, cloxacillin, or boronic acid (26).

In the mCIM, the test strain is incubated in a tube with a carbapenem disc at 35 °C for 4 h. Then the disc is placed on a plate pre-inoculated with a carbapenem-susceptible strain, and the plate is incubated for another 16–20 h. If there is no inhibition zone around the disc, the tested strain is positive for carbapenemases (54). The mCIM test does not require special reagents and media for implementation (55).

In contrast, the CarbaNP test is a biochemical test that detects carbapenem hydrolysis (phenol red dye changes to yellow due to a decrease in pH) (56). The CLSI recommends the use of this test when carbapenemase production is suspected in *Enterobacterales*, *Acinetobacter* spp., and *P. aeruginosa* (57). For these species, a high level of sensitivity (>90 %) and specificity (>90 %) is achieved for the detection of KPC, New Delhi metallo-beta-lactamases (NDM), imipenem-hydrolysing beta-lactamases (IMI), Verona integron-encoded metallo-beta-lactamases (VIM), and *Serratia marcescens* enzymes, while the sensitivity for detecting OXA-48 is low (<11 %) (58).

## MECHANISMS OF CARBAPENEM RESISTANCE

Carbapenems belong to beta-lactams, a group of antibiotics that inhibit bacterial cell wall formation by covalently binding to essential penicillin-binding proteins (55). The first beta-lactam antibiotic, benzylpenicillin, was discovered by Alexander Fleming in 1928, and many new ones have been discovered since. Today, there are several major classes of beta-lactams, usually classified as penicillins, cephalosporins, cephamycins, monobactams, and carbapenems. The first carbapenem discovered was thienamycin naturally produced by a soil bacterium *Streptomyces cattleya* (59, 60). Thienamycin exhibits strong antibacterial efficacy against gram-negative bacteria and is very stable even in the presence of certain beta-lactamase enzymes.

### Intrinsic and expected carbapenem resistance

Intrinsic resistance is unrelated to previous antibiotic exposure or non-sexual gene transfer (61). The genes for intrinsic resistance are usually located on the bacterial chromosome and shared by members of the same species or genus (62). Acquired resistance, on the other hand, is gained by bacteria through all possible routes of genetic material transfer, transformation, transposition, and conjugation, commonly referred to as horizontal gene transfer (HGT), with the most common route being the plasmid-mediated transmission.

As mentioned earlier, the first carbapenems were derived from *S. cattleya* of the *Streptomyces* genus. It was, therefore, safe to assume that at least *S. cattleya* and possibly many other streptomycetes would be intrinsically resistant to carbapenems. Interestingly, relevant scientific data on carbapenem resistance of streptomycetes are hard to find. One study by Lazim et al. (63) showed high *Streptomyces* sp. resistance to imipenem and meropenem, which the authors attributed to the production of metallo-beta-lactamase.

Another species with intrinsic carbapenem resistance is a gram-negative *Stenotrophomonas maltophilia*, also producing metallo-beta-lactamase (64). It has been considered an emerging pathogen of concern ever since it was isolated from hospitalised patients two decades ago (65). It has also been the most common cause of respiratory co-infections and bacteraemia during the COVID-19 pandemic (66). According to the literature, this bacterium is present in every possible environmental compartment (67).

Other environmentally ubiquitous species which can be intrinsically resistant to carbapenem are *Aeromonas* spp., as they contain the so called carbapenem-hydrolysing *Aeromonas* metallo-beta-lactamase (CphA) on the bacterial chromosome. These species have a great potential for spreading carbapenem resistance, especially in bodies of water (39).

Although clinically important bacteria are undoubtedly present in the environment, intrinsic resistance to carbapenems is not common among them, and most acquire resistance via HGT, usually through plasmid-mediated transmission or, rarely, mutation (64).

Closely related to intrinsic resistance is the term “expected resistant phenotype”. Since 2021, the EUCAST has decided to replace the term “intrinsic” with “expected”, as resistance may change with exposure to an antibiotic (68). For example, if an identified *K. pneumoniae* isolate turns out to be susceptible to ampicillin, its identification and/or susceptibility testing should be reviewed (69). Simply put, the expected resistant phenotype means that isolates of a species (or group of species) are generally and universally resistant, that is, that more than 90 % of all isolates, regardless of origin, exhibit a characteristic resistance mechanism or MIC values above the breakpoint listed in the EUCAST tables.

### Horizontal gene transfer and resistance acquisition

Horizontal transfer of genetic information between genomes is non-sexual, unlike that from parent to offspring. In the prokaryotic world, HGT is possible between different species, genera, orders, and even phyla and domains (70). It takes place in every environment, mainly where there are high concentrations of living cells, such as soil or WWTP, and especially in different kinds of biofilms (70). For example, the plasmid encoding OXA-48 carbapenem-resistant gene readily transfers from *E. cloacae* to other *Enterobacterales* in the gastrointestinal tract by conjugation (71). Conjugation is the gene transfer between bacteria in direct physical contact or through a bridge or tube-like connection between cells. Another mechanism of transfer is transformation, in which a recipient cell takes DNA floating around it and integrates it into its own genome.

Resistance acquired this way is common in many clinical species (70, 72). Indeed, a large body of evidence shows that HGT is responsible for the spread of antibiotic resistance in the environment (70, 73, 74), especially in biofilms, which significantly boosts gene transfer (75,76,77).

### Acquired carbapenem resistance

**Figure 3:**
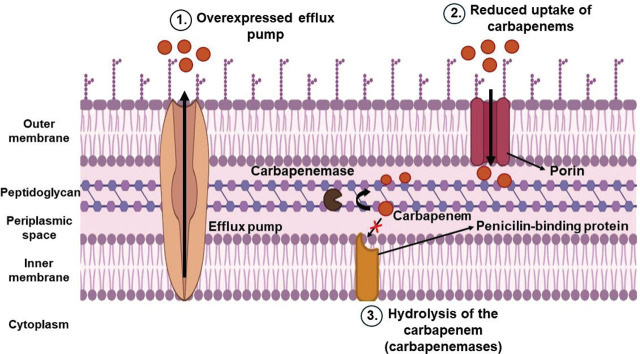
Mechanisms of carbapenem resistance in environmental isolates (drawing created with bioRender). 1) overexpression of the efflux pump; 2) reduced porin activity; 3) carbapenemase-catalysed reactions (carbapenem hydrolysis)

Resistance to carbapenems is mediated by the following mechanisms: reduced porin function, overexpression of efflux pumps, and enzyme-catalysed reactions (owed to carbapenemase production) (78) (Figure 3).

Porins are outer membrane proteins channelling antibiotics into the periplasmic space where penicillin-binding proteins are located, and their function is diminished by mutations, as reported by Buehrle et al. (79), who found that carbapenem resistance in *P. aeruginosa* was owed to porin mutations, production of beta-lactamases, and overexpression of efflux pumps (MexA-MexB-OprM).

Overexpression of efflux pumps, in turn, enables clearing of carbapenems from the cell, as reported for *A. baumannii*, in which the overexpression of the RND-type efflux pump AdeABC was observed (80).

The third major mechanism is the hydrolysis of carbapenems by specific beta-lactamases called carbapenemases (55). According to the Ambler classification, carbapenemases belong to three of the four classes (A, B, and D) (34). Classes A and D are called serine beta-lactamases, because they have a serine residue that cleaves the beta-lactam ring (80). Class A includes KPCs, Guiana extended spectrum (GES), and IMI (81). KPCs were first detected in *K. pneumoniae*, but they are now detected in other species such as *E. coli, Enterobacter*, *Citrobacter*, and *Serratia* sp. (80). Strains producing KPCs pose a significant threat, as they resist a broad spectrum of beta-lactams (55). Clinically relevant carbapenem-resistant strains are those producing KPC and NDM (82).

Class B carbapenemases are called metallo-beta-lactamases, most notably NDM and VIM, which contain zinc ions responsible for carbapenem hydrolysis (80). They have the ability to degrade a wide range of beta-lactams, except monobactams (55).

Class D refers to oxacillinases (OXA), which are common in *A. baumannii* (80), and the first OXA type described in *A. baumannii* was OXA-23 (83). The genes for OXA-23 are located on plasmids and are most commonly found in species of the *Acinetobacter* genus, although they have also been detected in CRE species. In addition to hydrolysing carbapenems, OXA-23-like enzymes can also hydrolyse antibiotics from other classes, such as penicillins and cephalosporins. The presence of OXA-23 alone is sufficient to confer carbapenem resistance, but the overexpression of AdeABC efflux pumps leads to more pronounced resistance (increased MIC values) (83). A similar mechanism applies to the OXA-40 enzyme, which has been detected in *Acinetobacter*, *K. pneumoniae*, and *P. aeruginosa*. In *A. baumannii*, the OXA-51-like genes are located on the chromosome and express activity against carbapenems only when the ISA*ba1* gene is positioned upstream (83,84,85).

Carbapenemase-producing genes *KPC*, *VIM*, *NDM*, and *OXA-48* are plasmid-mediated, whereas *IMI* is located on the chromosome and plasmid (81). Genes encoding carbapenemases found in clinical isolates have also been identified in environmental isolates. Jin et al. (86) reported KPC-producing *K. pneumoniae* harbouring the *bla*_KPC-2_ gene isolated from the influent and effluent of hospital sewage. Another example is CRAB isolated from swine manure containing the *bla*_OXA-23_. This suggests that the mechanisms of resistance to carbapenems are likely similar in both clinical and environmental isolates (38).

## ENVIRONMENTAL DISSEMINATION OF CARBAPENEM-RESISTANT BACTERIA

CRB species are now reported in hospital settings and in every environmental compartment worldwide (Table 2). However, pathways of entry and spread of CRB from infected patients to the environment are still insufficiently evidenced.

**Table 2:** Literature reports of CRB presence in different environmental samples

**Species**	**Sample type**	**Reference**
*A. baumannii, Enterobacterales*	Hospital environment (sheets, bed rail, bedside table, keyboard, devices, ventilator tube, air sample, air conditioner, sheets and infusion pump)	(88,89,90, 92, 93)
*K. pneumoniae, A. baumannii, P. aeruginosa*	Hospital sewage	(44, 95, 96)
*K. pneumoniae, A. baumannii, P. aeruginosa*	Urban sewage	(96, 97)
*K. pneumoniae, A. baumannii, Pseudomonas* sp.	River water and sediment	(30, 32, 104, 126)
*Klebsiella* spp., *Acinetobacter* spp., *Pseudomonas* spp. *E. coli*	Sea water and sediment	(109, 110)
*E. asburiae*	Lake water	(108)
*A. baumannii*	Soil	(111)
*K. pneumoniae, A. baumannii, E. cloacae*	Hospital WWTP	(98, 102)
*K. pneumoniae, A. baumannii, E. coli*	Urban WWTP	(43, 100)
*A. baumannii, E. coli*	Companion animals	(113, 115)
*K. pneumoniae*	Domestic animals	(118)
*K. pneumoniae, A. baumannii, E. coli*	Ready-to-eat vegetables	(121, 122)

### Carbapenem-resistant bacteria in hospital environment

The hospital environment is a common source of CRB infections. According to the latest US CDC data, the rate of hospital-onset CRAB and CRE infections increased between 2019 and 2022 (87). Many hospital outbreaks have been reported since the past decade, and nosocomial infections with CRE are considered endemic in certain areas. CRAB strains have been isolated from many patient samples (blood, sputum, urine) but have also been detected in hospital beds, devices, and similar surfaces. In one study (88), all CRAB detected on surfaces such as the bed rail, bedside table, keyboard, and cardiograph monitor panel formed the same cluster with the CRAB isolate from the sputum a patient. In a similar study (89), CRE recovered from various surfaces (bedside table, sheet surfaces around the pillow, crotch and legs, and infusion pump) pointed to the patients with infections caused by KPC-producing CRE.

If treatment requires the use of invasive devices such as ventilators and catheters, the risk of infection increases. For example, CRAB ST499^P^ (Pasteur’s scheme) containing the *bla* was isolated from a ventilator tube, while CRAB ST2^P^ with the *bla*_OXA-23_ and *bla*_NDM-1_ was isolated from an infusion pump (90). In one study (91), portable machines such as Doppler ultrasound and ECG were inoculated with a DNA marker to determine the route for dissemination of CRB. A few days after inoculation, surfaces in patient environment and other portable devices were sampled, and the findings confirmed contamination and indicated healthcare workers’ hands as the likely route of dissemination.

Although data are still limited, some studies even point to air contamination, as samples taken from the air surrounding CRAB-infected patients with respiratory condition tested positive for CRAB (92). More precisely, the positive findings were much more common during patient care (such as open-system endotracheal suctioning, closed-system endotracheal suctioning, or changing bed sheets) than during rest. In another study (93), CRAB ST208^Ox^ (Oxford’s scheme) isolated from hospital patients was identical to the isolate recovered from the air conditioner.

### Carbapenem-resistant bacteria in hospital and urban sewage

High presence of CRB in the hospital environment seems to overflow into hospital waste, which is considered a hot spot for CRB. This calls for efficient waste treatment to prevent CRB dissemination into the natural environment. Many studies have evidenced the presence of CRB in hospital sewage, such as *K. pneumoniae* ST258 (PubMLST scheme) harbouring *bla*_KPC-2_, which is considered an epidemic high-risk clone (44). Sixteen isolates (*K. pneumoniae* ssp. *pneumoniae*, *Enterobacter cloacae* complex, *K. pneumoniae* ssp. *ozaenae*, *Klebsiella oxytoca*, *Citrobacter farmer* and *Kluyvera intermedia*) producing class A or D carbapenemases were found in hospital sewage in Brazil, and all were carriers of *bla*_KPC-2_ (94). However, this study did not compare clinical isolates and those found in hospital sewage to confirm their connection. A study that compared clinical isolates with those found in sewage discharged from two long-term care facilities evidenced dissemination of OXA-23-producing *A. baumannii* from patients to nursing home sewage (95).

Urban sewage collects household, industrial, and hospital wastewater. One study (96) identified carbapenem-resistant *K. pneumoniae* and *P. aeruginosa* in samples taken directly from the inlet pipe of a sewage treatment plant. Untreated or improperly treated sewage waste discharged into the environment increases the risk of entry and spread of CRB in the environment, as evidenced by a study in which samples of untreated wastewater and river water before and after discharge of untreated wastewater were analysed (97). River water before wastewater discharge was CRAB-free, while all other samples contained CRAB.

### Carbapenem-resistant bacteria in wastewater treatment plants (WWTP)

Many studies have shown that WWTP enable bacteria to multiply, survive, and spread into the environment. One study (98) has shown that the proportion of clinically relevant species in the effluent of WWTP receiving hospital and urban sewage was significantly higher than in the influent. In another study (99), CRAB were found in all stages of wastewater treatment except in the lime-sterilised sludge. Furthermore, some of the *A. baumannii* isolates belonging to the International Clonal Lineage 2 (IC2) were closely clustered despite being collected at different times and stages, which suggests that CRAB are continuously introduced through urban wastewater and consistently present in the influent. In another study (100), a carbapenem-resistant *E. coli* strain (ST746) with the *bla*_NDM-5_ gene was found in the influent of urban WWTP, but effluents were not sampled to know if the same strain was present in the final treatment stage. A WWTP effluent study (43) found *A. baumannii*, *E. coli*, and *K. pneumoniae* carrying several acquired resistance genes such as *bla*_OXA-24_ (*A. baumannii*) and *bla*_KPC-3_ (*E. coli* and *K. pneumoniae*). In contrast, another study (101) identified intrinsically carbapenem-resistant *Citrobacter* sp., *Enterobacter* sp., *Leclercia* sp., and *Lelliota* sp. in raw hospital wastewater and urban WWTP but not in the final effluent after UV disinfection, indicating the effectiveness of bacterial removal. In a study of WWTPs receiving hospital and urban sewage across the US, Mathys et al. (102) observed a difference in the proportion of clinically relevant species in effluents between WWTPs located in urban areas (50 %) and rural areas (8 %). The detected species included *E. cloacae* and *K. pneumoniae*, with the dominant carbapenemase-encoding gene among the found species being the *bla*_KPC-2_, which is also the most common gene in isolates responsible for infections in the USA. They noted a difference in the disinfection efficiency of the effluent and highlighted the potential advantage of UV over chlorination.

Many studies have refuted the notion that the presence of antibiotics enables the selection or survival of CRB. Bengtsson-Palme et al. (103) reported no impact of selective pressure from different classes of antibiotics on the composition of antibiotic-resistant genes in hospital and urban WWTPs. A concerning finding was the detection of the *bla*_OXA-48_ in surplus and digested sludge, which had not been detected in the primary sludge. The authors observed that a substantial change in the bacterial communities occurred already in the sewage pipes.

### Carbapenem-resistant bacteria in water bodies and soils

**Figure 4:**
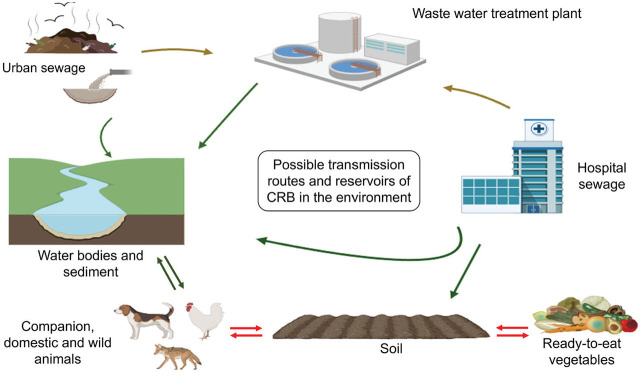
Possible transmission routes and reservoirs of CRB in the natural environment (drawing created with bioRender). 1) discharge of untreated hospital and urban sewage; 2) hospital and urban sewage that goes through WWTP but is released as partially treated effluent; 3) transmission of CRB through animals and ready-to eat-vegetables

Rivers are considered hot spots for CRB bacteria, mainly because they receive urban and hospital sewage and effluents from WWTPs (Figure 4). Lepuschitz et al. (104) found two isolates of carbapenem-resistant *K. pneumoniae* ST11 and ST985 in samples taken from two city rivers receiving WWTP effluents but not in samples taken upstream of the WWTPs. These isolates showed a high degree of similarity to the corresponding clinical isolates from hospitals in the respective cities. Another study (105) reported *K. pneumoniae*, *Enterobacter*, and *Citrobacter* harbouring *bla*_KPC-3_, *bla*_NDM-1_, and *bla*_GES-5_ encoding genes in river samples collected near pig farming facilities, which could have influenced the introduction of these species into the river. However, the impact of pig farming facilities still needs to be confirmed. A similar study (30) showed the presence of *Pseudomonas* sp. resistant to ertapenem and imipenem in a river, with identified *bla*_VIM-2_ gene. The highest proportion of imipenem-resistant bacteria was found at a sampling site where the water quality was poor due to high concentrations of ammonia and phosphorus. Other studies found *K. pneumoniae* subsp. *pneumoniae* (*bla*_VIM_), carbapenem-resistant *Acinetobacter* spp., and CRAB in rivers (32, 106, 107).

Only a few studies investigated the presence of CRB species in lake and sea water. Harmon et al. (108) found carbapenem-resistant *E. asburiae*, along with intrinsically resistant *S. maltophilia*, and *Aeromonas veronii* in a lake, while Paschoal et al. (109) reported carbapenem-resistant species *Acinetobacter* spp. (*bla*_OXA-23_), *Klebsiella* spp. (*bla*_KPC-2_), and *Pseudomonas* spp. (*bla*_VIM-2_) in sea water samples taken from urban beaches.

Some studies extended their research to river bed sediments. One (32) reported CRB in samples of river water and sediment and revealed a negative correlation between CRB and intestinal enterococci, suggesting CRB were not necessarily introduced into the environment through faeces. Another study (110) found carbapenem-resistant *E. coli* in sediment along the coast in port and industrial zones, suggesting that the spread likely came from untreated waste.

Research of soil is still scarce but confirms the presence of CRB species, most likely owed to illegal waste or wastewater disposal. One study (111) found CRAB ST231_Ox_ and ST195_Ox_ in soil samples taken from an illegal dump site. Given that isolates exhibited characteristics of clinical isolates, the authors assumed that the dump site received hospital waste. Another study (112) reported the presence of *bla*_KPC_, *bla*_NDM_, and *bla*_VIM_ in soil taken at broiler, layer, and pig farms.

### Carbapenem-resistant bacteria in animals and ready-to-eat vegetables

The One Health approach starts from the premise that the health of humans, animals, plants, and the wider environment (including ecosystems) is closely linked and interdependent (8). To effectively combat antibiotic resistance, it is essential to thoroughly investigate whether CRB species truly pose a public health risk through potential transmission between these components.

Many studies set out to determine potential CRB transmission between animals and humans and whether isolates found in animals were related to those found in humans. A study of samples taken from dogs’ ears showed that the carbapenem-resistant *E. coli* ST167 isolate, positive for the NDM-5 gene, was also found in samples taken from their owners (113). However, the study could not determine which of the two was the initial carrier or whether the dogs became infected at a veterinary hospital.

Staying in healthcare facilities poses a risk of infection for both humans and animals. In one study (114), 13 animals were found to carry CRB, of which 12 had previously been hospitalised. Four animals harboured extensively drug-resistant ST2^P^ or ST451^Ox^ *A. baumannii*. Whole-genome MLST showed a high correlation between IC2 isolates, which are often the cause of hospital outbreaks. Another study (115) found three CRAB isolates positive for OXA-23 in dogs and cats from veterinary clinic. The CRAB isolates from dogs were ST1^P^/ST231^Ox^ and IC1, while the isolates from cats were ST10^P^/ST585^Ox^ and IC8.

One study (116) stated that bovine isolates of *A. baumannii* are more similar to environmental strains and isolates from wild animals than to those found in humans and companion animals. Another study (117) identified carbapenem-resistant *E. coli* positive for NDM-1 in a pygmy sperm whale, which, according to phylogenetic analysis, clustered with the poultry and human ST162. In an Egyptian study (118), carbapenem-resistant *K. pneumoniae* harbouring *bla*_KPC_, *bla*_OXA-48_, or *bla*_NDM_ was found in samples collected from broiler farm workers (five isolates), broiler chickens (15 isolates), and drinking water (three isolates) at the farm. However, the authors did not run MLST to trace transmission between poultry and humans, that is, to find if the same ST was present across samples. In another study (119), *A. baumannii* isolates from avian wildlife (white stork) did not cluster with human lineages, but avian livestock (chicken and geese) isolates clustered with IC7 and IC8. This points to different dissemination routes of CRB species between wild and domestic animals.

Another potential source of CRB are fresh, untreated vegetables, and the pathways of contamination may vary. For instance, organic farming fertilises soil with manure, which may contain CRB and contaminate the soil, plants, and, ultimately, the food chain. One such contamination was reported for swine manure samples identified with IC2 and ST195^Ox^ CRAB isolates carrying acquired *bla*_OXA-23_ (38). Another route can be contaminated water used for agricultural purposes (120).

Vegetable contamination (namely, cucumber, lettuce, and curly endive) was reported by Jiménez-Belenguer et al. (121), who identified the isolates of *E. coli* (*bla*_NDM-1_) and *K. pneumoniae* (*bla*_KPC-2_). The *E. coli* isolates shared the same pulsed-field gel electrophoresis (PFGE) pattern and ST. *K. pneumoniae* ST23^P^ isolates also exhibited identical PFGE patterns on the same type of vegetable. In another study (122), two CRAB isolates were detected on organic vegetables, but no MLST typing was done to establish a link with human/hospital isolates.

### Survival of carbapenem-resistant bacteria in environmental conditions

Once CRB enter the environment, their survival depends on environmental conditions. Certain strains of *A. baumannii* can long persist on various surfaces, which can be important in hospital settings. One study (123) showed that biofilm-forming strains of *A. baumannii* survived for up to 35 days on glass surfaces under dry conditions.

Data on the survival of CRB species in anaerobic conditions are scarce. One study (98) reported significantly higher proportion of CRAB in digested sludge than in WWTP influent. The reason may be incorporation of CRAB into flocs of sludge that undergoes anaerobic mesophilic digestion, which may enable the proliferation of these species. Another study (99) also reported CRAB isolated from digested sludge, suggesting that CRAB can survive in anaerobic conditions.

In aerobic conditions, CRAB isolates recovered from WWTP effluent were reported to have survived and even multiplied for 50 days, once they were moved to and cultivated in sterilised WWTP effluent (33). Clinical and environmental *K. pneumoniae* ST147 showed similar behaviour in ultra-pure water and non-sterile urban runoff water (124). During the eight-day incubation, the abundance of both isolates remained unchanged in sterile pure water, while their numbers decreased by a 3-log in urban runoff water due to competition with other species.

The survival of environmental and clinical isolates of CRAB in soil under laboratory conditions depended on the pH value and moisture of the soil. Dekić et al. (125) reported that the species could survive for up to five months in alkaline soil and that their numbers dropped with lower moisture content, while their survival was only an hour in an acidic medium (pH 2.51).

## CONCLUSION

Numerous studies have confirmed that CRB of clinical relevance are present in hospital and natural environments all over the world and that their presence in the environment seems to be linked to anthropogenic sources.

In seeking potential transmission routes between humans, animals, and the environment, One Health approach determines the species at the ST level, phenotypic antibiotic susceptibility with a focus on acquired as opposed to intrinsic resistance, and mechanisms underlying carbapenem resistance. Species ST is a critical piece of information, as it enables linking clinically relevant and environmental CRB species. Currently, this approach is limited to the species, and further evidence is needed to see if the linkage is also valid at the strain level.

The issue of global antibiotic resistance should not be oversimplified and considered one-dimensional. It is far more reasonable to assume that resistance arises from tremendously complicated interactions between numerous bacteria from all environmental compartments. Only by correctly defining the problem can we find the way to slow down the spread of carbapenem resistance.
